# Proposed Role for COUP-TFII in Regulating Fetal Leydig Cell Steroidogenesis, Perturbation of Which Leads to Masculinization Disorders in Rodents

**DOI:** 10.1371/journal.pone.0037064

**Published:** 2012-05-17

**Authors:** Sander van den Driesche, Marion Walker, Chris McKinnell, Hayley M. Scott, Sharon L. Eddie, Rod T. Mitchell, Jonathan R. Seckl, Amanda J. Drake, Lee B. Smith, Richard A. Anderson, Richard M. Sharpe

**Affiliations:** 1 MRC Centre for Reproductive Health, The Queen's Medical Research Institute, The University of Edinburgh, Edinburgh, United Kingdom; 2 Endocrinology Unit, University/BHF Centre for Cardiovascular Science, The Queen's Medical Research Institute, The University of Edinburgh, Edinburgh, United Kingdom; University of Muenster, Germany

## Abstract

Reproductive disorders that are common/increasing in prevalence in human males may arise because of deficient androgen production/action during a fetal ‘masculinization programming window’. We identify a potentially important role for Chicken Ovalbumin Upstream Promoter-Transcription Factor II (COUP-TFII) in Leydig cell (LC) steroidogenesis that may partly explain this. In rats, fetal LC size and intratesticular testosterone (ITT) increased ∼3-fold between e15.5-e21.5 which associated with a progressive decrease in the percentage of LC expressing COUP-TFII. Exposure of fetuses to dibutyl phthalate (DBP), which induces masculinization disorders, dose-dependently prevented the age-related decrease in LC COUP-TFII expression and the normal increases in LC size and ITT. We show that nuclear COUP-TFII expression in fetal rat LC relates inversely to LC expression of steroidogenic factor-1 (SF-1)-dependent genes (*StAR*, *Cyp11a1*, *Cyp17a1*) with overlapping binding sites for SF-1 and COUP-TFII in their promoter regions, but does not affect an SF-1 dependent LC gene (*3β-HSD*) without overlapping sites. We also show that once COUP-TFII expression in LC has switched off, it is re-induced by DBP exposure, coincident with suppression of ITT. Furthermore, other treatments that reduce fetal ITT in rats (dexamethasone, diethylstilbestrol (DES)) also maintain/induce LC nuclear expression of COUP-TFII. In contrast to rats, in mice DBP neither causes persistence of fetal LC COUP-TFII nor reduces ITT, whereas DES-exposure of mice maintains COUP-TFII expression in fetal LC and decreases ITT, as in rats. These findings suggest that lifting of repression by COUP-TFII may be an important mechanism that promotes increased testosterone production by fetal LC to drive masculinization. As we also show an age-related decline in expression of COUP-TFII in human fetal LC, this mechanism may also be functional in humans, and its susceptibility to disruption by environmental chemicals, stress and pregnancy hormones could explain the origin of some human male reproductive disorders.

## Introduction

Phenotypic masculinization is a pivotal event in mammalian development, diverting the fetus from the female ‘set-up’ programme of development. The key driver of this process is testosterone produced by the fetal Leydig cells (LC) [1]. This occurs early in fetal development, immediately after testis differentiation, in what has been termed the masculinization programming window (MPW; e15.5–e18.5 in the rat) [1], [2]. The level of androgen production/action in the MPW critically determines later reproductive development and final size of all male reproductive organs in the rat [2], [3], [4], [5], although androgen production after the MPW is important for reproductive organ differentiation and growth [1], [4], [5]. Deficiency in androgen production/action within the MPW results in ‘testicular dysgenesis syndrome (TDS)’ disorders such as hypospadias, cryptorchidism and reduced testis size/sperm production in rats [1], [3], [4], [5] and humans [6], [7], [8].

**Figure 1:**
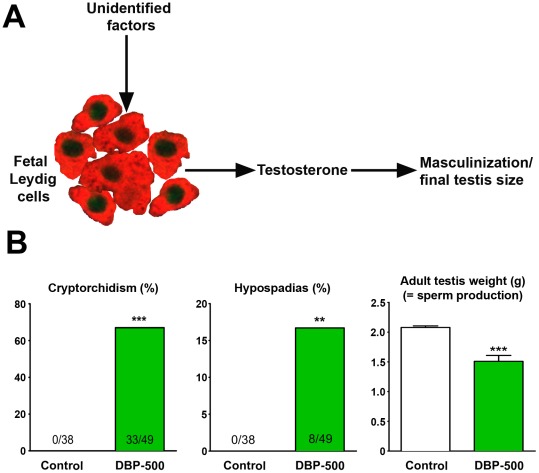
Critical importance of local (unknown) factors in the regulation of testosterone production by fetal Leydig cells in the rat during the masculinization programming window (A) and downstream effects of impairment of testosterone production by fetal exposure to dibutyl phthalate (DBP; 500 mg/kg/day from e13.5–e21.5) (B).

Therefore, regulation of testosterone production by fetal LC within and after the MPW is fundamentally important for normal male development, yet the mechanisms involved are largely unknown (Fig. 1A). In rodents, it has been presupposed that unknown paracrine mechanisms stimulate LC steroidogenesis during this period, as secretion of the main physiological LC stimulator, luteinizing hormone (LH), does not begin until after the MPW, and knockout of either *LHβ* or its receptor does not impair masculinization [1]. In contrast, in humans/primates, inactivating mutations of the LH receptor, although not of LH, impair masculinization [1]. This is because the primate placenta produces an LH-like chorionic gonadotropin (CG) that stimulates fetal LC, whereas the rodent placenta does not [1]. However, even in humans, the balance of evidence points to CG not being the sole driver of fetal LC steroidogenesis during the presumptive MPW, so that (unidentified) local stimulatory factors may also play a role [1]. Consequently, ignorance about the regulation of fetal testis steroidogenesis in and around the MPW is a major obstacle to identifying how normal masculinization is driven and what can impact this to induce TDS/masculinization disorders.

We have developed an animal model for TDS involving fetal exposure to the ubiquitous environmental chemical, dibutyl phthalate (DBP) [9]. Exposure of pregnant rats to DBP suppresses fetal LC steroidogenesis before and after the MPW because of the down-regulation of several genes that play critical roles in LC steroidogenesis, all of which are regulated by steroidogenic factor 1 (SF-1) [10], [11], [12], [13], [14], [15]. However, SF-1 expression itself is unaffected by DBP exposure and not all SF-1-dependent genes expressed in the fetal testis are impacted [12]. Whilst searching for an explanation for the latter observations, we identified an important role for chicken ovalbumin upstream promoter transcription factor II (COUP-TFII; also known as NR2F2). COUP-TFII is a widely expressed orphan nuclear receptor of the steroid/thyroid family [16]. Moreover, prepubertal deletion of COUP-TFII in the mouse results in failure of adult LC to differentiate, leading to near absence of testosterone production and infertility [17], implying a key role in (adult) LC development. The initial aim of the present studies was to evaluate if a mechanism involving COUP-TFII expression in fetal LC could explain the steroidogenic effects of DBP exposure in the rat, which our findings suggest it does. However, in so doing, wider implications emerged, namely evidence that regulation of LC steroidogenesis in and after the MPW may not be under stimulatory control by paracrine mechanisms, but rather may be actively repressed (by COUP-TFII) and that lifting of this repression is crucial for expansion of LC steroidogenic function during and after the MPW. We show that various treatments that impair fetal LC steroidogenesis in rats all maintain or induce COUP-TFII expression in fetal LC, and that prevalence of COUP-TFII expression in fetal LC in rats and mice is inversely related to ITT in every situation investigated. Vulnerability of this local mechanism to disruption by endogenous and exogenous factors could potentially explain why disorders (TDS) stemming from mild deficiency in androgen action in the MPW are common in humans.

## Results

### DBP exposure of fetal rats and the induction of later TDS disorders

To characterize the relationship between COUP-TFII and steroidogenesis, we initially utilized an established DBP treatment regime (500 mg/kg/day from e13.5–e21.5) that induces a major reduction in intratesticular testosterone (ITT) at e21.5 (see below), leading to a high incidence of TDS-like disorders in adulthood (Fig. 1B).

### DBP effects on intratesticular testosterone, LC number and nuclear/cytoplasmic volume

**Figure 2:**
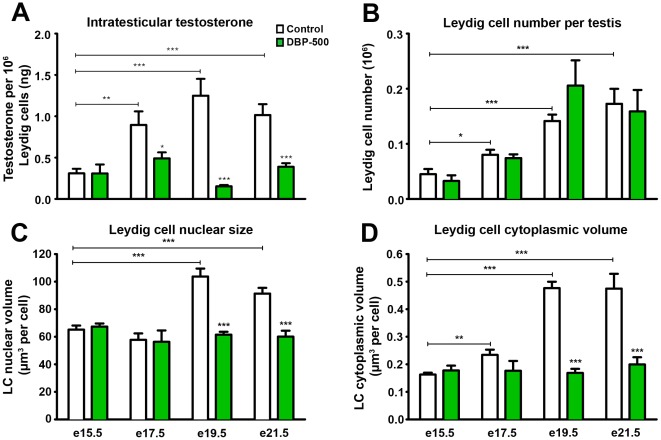
Effect of *in utero* exposure of rats to vehicle (control) or dibutyl phthalate (DBP: 500 mg/kg/day) on age-dependent changes in intratesticular testosterone levels per 10^6^ fetal fetal Leydig cells (A), Leydig cell number per testis (B), Leydig cell nuclear volume (C) and Leydig cell cytoplasmic volume (D). Values in A are Means ± SEM for 5–12 animals at each age (minimum of 3 litters per group). Values in B–D are Means ± SEM for 4–8 animals in each group (minimum of 3 litters per group). *p<0.05, **p<0.01, ***p<0.001, in comparison with respective controls; other comparisons are indicated by capped lines.

In control animals, intratesticular testosterone (ITT), corrected for the number of LC per testis (see below), significantly and progressively increased between e15.5 and e19.5/e21.5. No such increase occurred in testes of DBP-exposed animals (Fig. 2A). We performed stereological quantification of LC number (Fig. 2B) and cytoplasmic volume per LC (Fig. 2D), which both increased ∼3-fold between e15.5 and e21.5 in control animals with a smaller increase in LC nuclear volume (Fig. 2C). DBP exposure had no effect on the number of fetal LC at any age (Fig. 2B), but prevented the normal age-dependent increase in LC cytoplasmic volume (Fig. 2D) and nuclear volume (Fig. 2C). The increase in LC cytoplasmic volume in controls between e15.5 and e21.5 and its prevention by DBP exposure (Fig. 2D) paralleled the observed changes in ITT (Fig. 2A).

### DBP-exposure down-regulates mRNA expression of selected SF-1-regulated steroidogenic genes

**Figure 3:**
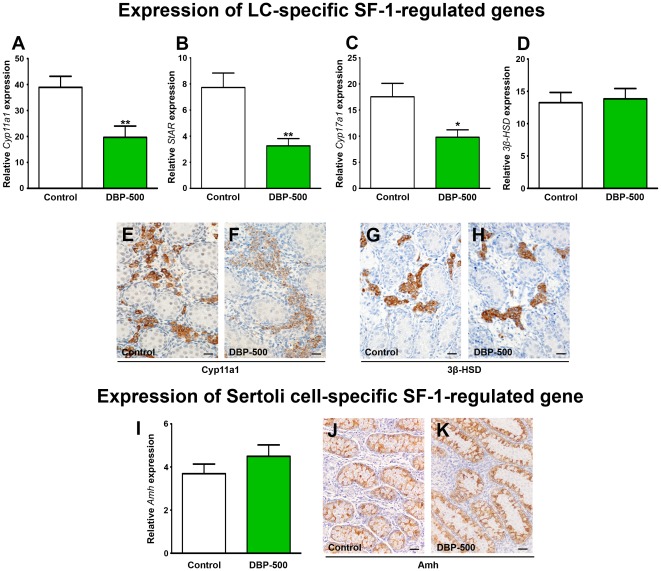
Effect of *in utero* exposure of rats to vehicle (control) or dibutyl phthalate (DBP: 500 mg/kg/day) on steroidogenic enzyme and anti-Müllerian hormone gene expression in testes at e21.5. (A) cytochrome P450, family 11, subfamily a, polypeptide 1 (*Cyp11a1*), (B) Steroidogenic acute regulatory protein *(StAR*), (C) cytochrome P450, family 17, subfamily a, polypeptide 1 (*Cyp17a1*), (D) hydroxy-delta-5-steroid dehydrogenase, 3 beta- and steroid delta-isomerase 1 (*3β-HSD*), (I) Anti-Müllerian hormone (*Amh*). Values are Means ± SEM for 19–22 animals per group (minimum of 5 litters per group). *p<0.05, **p<0.01, in comparison with respective control. (E–F) Immunohistochemistry for Cyp11a1 on e21.5 testis sections isolated from control (E) and DBP-500-exposed (F) animals. (G–H) Immunohistochemistry for 3β-HSD on e21.5 testis sections isolated from control (G) and DBP-500-exposed (H) animals. (J–K) Immunohistochemistry for Amh on e21.5 testis sections isolated from control (J) and DBP-500-exposed (K) animals. Scale bars E–H = 20 µm.

Previous studies have demonstrated the suppression of steroidogenic enzyme gene expression, such as *StAR*, *Cyp11a1* and *Cyp17a1*, in the fetal rat testis after exposure to DBP [10], [11], [12], [13], [14], [15]. We therefore used quantitative real-time PCR to evaluate steroidogenic enzyme gene expression in e21.5 control and DBP-exposed animals, when ITT suppression is maximal (Fig. 2A). Exposure to DBP reduced the mRNA expression of *Cyp11a1*, *StAR* and *Cyp17a1* (Figs. 3A–C), whereas mRNA expression for *3β-HSD* was unaffected (Fig. 3D). We have previously published that these genes are all SF-1-regulated [12] and analysis of their promoter regions revealed that the DBP-affected genes (*Cyp11a1*, *StAR* and *Cyp17a1*) all have overlapping SF-1/COUP-TFII binding sites in their promoters, whereas *3β-HSD* only has an SF-1 binding site (Table 1). Another gene with SF-1 and COUP-TFII binding sites, but expressed in Sertoli cells is *Amh* (Table 1). Exposure to DBP did not affect *Amh* mRNA expression (Fig. 3I). These gene expression studies utilized whole testes, but the genes analyzed are cell-specific in the fetal testis, as confirmed by immunoexpression studies comparing Cyp11a1 (Figs. 3E,F), 3β-HSD (Figs. 3G,H) and Amh (Figs. 3J,K), which also confirmed selective suppression by DBP of Cyp11a1 but not the latter two.

**Table 1:** An overview of SF-1, COUP-TFII and SF-1/COUP-TFII binding sites in the promoters of *StAR*, *Cyp11a1*, *Cyp17a1*, *Hsd3b1* and *Amh*.

		Promoter binding sites (bp downstream of transcription start site)		
Gene name	NCBI accession number	SF-1 binding site	COUP-TFII binding site	SF-1/COUP-TFII binding site
StAR	NM_031558	−150	−90*−110	−460−640−760−1330
Cyp11a1	NM_017286	-	-	−50−1840
Cyp17a1	NM_012753	−57	−278*	−3859
Hsd3b1 (3β-HSD)	NM_008293	−1530	-	-
Amh	NM_012902	−2529	−2325	-

* : “weak” COUP-TFII binding site.

StAR = steroidogenic acute regulatory protein; Cyp11a1 = cytochrome P450, family 11, subfamily a, polypeptide 1; Cyp17a1 = cytochrome P450, family 17, subfamily a, polypeptide 1; Hsd3b1 = hydroxy-delta-5-steroid dehydrogenase, 3 beta- and steroid delta-isomerase 1; Amh = anti-Müllerian hormone; SF-1 = steroidogenic factor-1; COUP-TFII = chicken ovalbumin upstream promoter transcription factor II; bp = basepair.

### Dose-dependent effects of DBP on COUP-TFII expression and ITT in fetal LC

**Figure 4:**
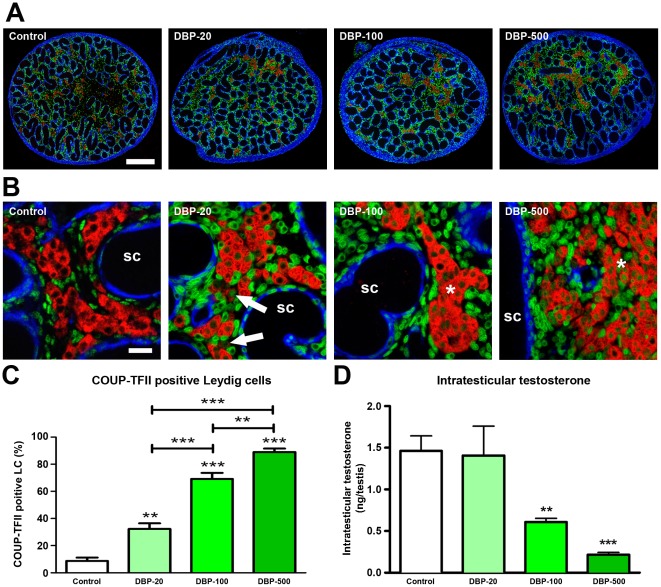
Altered COUP-TFII expression in fetal rat Leydig cells after *in utero* exposure to vehicle (control) or to different doses of dibutyl phthalate (DBP) and the relationship to intratesticular testosterone levels at e21.5. (A–B) Triple immunofluorescence for SMA (blue), 3β-HSD (red) and COUP-TFII (green) on testis sections from representative vehicle (control) and DBP-exposed animals. Note that major persistence of COUP-TFII expression in fetal Leydig cells is observed after exposure to DBP-100 and DBP-500 (100 and 500 mg/kg/day, respectively), whereas DBP-20 (20 mg/kg/day) had a much smaller effect. Asterisks indicate Leydig cell aggregates that are predominantly immunopositive for COUP-TFII. SC = seminiferous cords. Scale bar A = 200 µm, B = 20 µm. (C) Quantification of the percentage of COUP-TFII positive fetal Leydig cells in vehicle (control) and DBP-exposed animals using tiled high resolution images as shown in panel A. Values are Means ± SEM for 6–9 animals per treatment group (minimum of 3 litters per group). **p<0.01, ***p<0.001, in comparison with respective control; other comparisons are indicated by capped lines. (D) Corresponding intratesticular testosterone levels for the treatment groups in panels A and B. Values are Means ± SEM for 4–21 animals per group (minimum of 3 litters per group). **p<0.01, ***p<0.001, in comparison with respective control.

Since the LC-expressed genes down-regulated after DBP-exposure all have overlapping SF-1/COUP-TFII binding sites in their promoters, we studied the expression of COUP-TFII in testes of control and DBP-exposed animals. Pregnant female rats were treated with different doses of DBP (20, 100 and 500 mg/kg/day) and the testes were examined at e21.5 (Fig. 4). Real-time PCR analysis of *COUP-TFII* mRNA expression in whole fetal testes showed no change between control and DBP-exposed animals (Fig. S1), presumably because COUP-TFII is abundantly expressed in several cell types in the fetal testis other than the fetal LC (see below). We therefore utilized confocal microscopy and high resolution tiled images of complete testis cross-sections to investigate LC-specific COUP-TFII expression at e21.5 (Fig. 4A), which we quantified stereologically (Fig. 4B,C). Most fetal LC in fetuses exposed to 20 mg/kg/day DBP (DBP-20) were COUP-TFII immunonegative at e21.5, as in controls, whereas in fetuses exposed to 100 or 500 mg/kg/day (DBP-100 and DBP-500, respectively) COUP-TFII was expressed in ∼70% and ∼85% of fetal LC nuclei respectively (Fig. 4B,C). This dose-dependent effect of DBP on the percentage of fetal LC expressing nuclear COUP-TFII was inversely related to suppression of ITT (Fig. 4D). Some non-Leydig interstitial cells appeared to express COUP-TFII in their cytoplasm rather than the nucleus (Fig. 4B). However, this is a processing artifact that results in aberrant nuclear morphology (such that a central intranuclear vacuole appears to be the nucleus), as illustrated by comparing COUP-TFII expression with that of the nuclear-specific counterstain (DAPI; Fig. S2), confirming that COUP-TFII expression is confined to the nucleus in all cells in which it is expressed in the fetal testis.

### Age-dependent switching off of COUP-TFII nuclear immunoexpression in fetal LCis prevented by DBP-exposure

**Figure 5:**
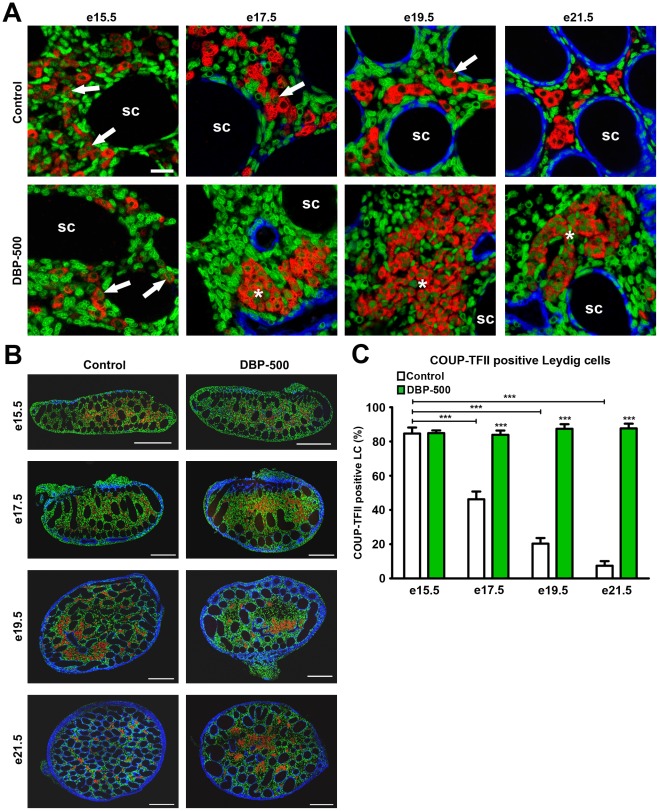
Age-dependent alteration in COUP-TFII expression in fetal rat Leydig cells in vehicle-exposed control rats and after *in utero* exposure to dibutyl phthalate (DBP; 500 mg/kg/day). (A–B) Triple immunofluorescence for SMA (blue), 3β-HSD (red) and COUP-TFII (green) on fetal testis sections from vehicle (control) and DBP-exposed animals. Arrows in A indicate examples of individual Leydig cells positive for COUP-TFII whereas asterisks indicate DBP-induced aggregates of Leydig cells which are predominantly COUP-TFII-immunopositive. SC = seminiferous cords. Scale bar A = 20 µm, B = 200 µm. (C) Quantification of the percentage of COUP-TFII positive fetal Leydig cells in vehicle (control) and DBP-exposed animals using tiled high resolution images as shown in panel B. Values are means ± SEM for 5–8 animals at each age (minimum of 3 litters per group). ***p<0.001, in comparison with respective control; other comparisons are indicated by capped lines.

We then examined COUP-TFII expression in fetal LC during normal development and how this was affected by exposure to 500 mg/kg/day DBP. At e15.5, testes from both control and DBP-exposed fetuses expressed nuclear COUP-TFII in >85% of fetal LC (3β-HSD immunopositive cells) (Fig. 5A,C). From e17.5 onwards in controls, the percentage of LC expressing nuclear COUP-TFII progressively declined, such that by e21.5<10% of LC were COUP-TFII-immunopositive. This correlated inversely to the significant and progressive increase in ITT between e15.5 and e19.5/e21.5 in control animals (Fig. 2A). In contrast, in DBP-exposed animals, COUP-TFII expression in fetal LC persisted unchanged from e15.5 to e21.5 (Fig. 5A,C), which correlated with prevention of the normal ITT increase after DBP-exposure (Fig. 2A). COUP-TFII expression in interstitial cells other than fetal LC did not change detectably with fetal age or DBP treatment.

### Late gestational exposure to DBP re-induces COUP-TFII nuclear expression in fetal LC and reduces ITT

**Figure 6:**
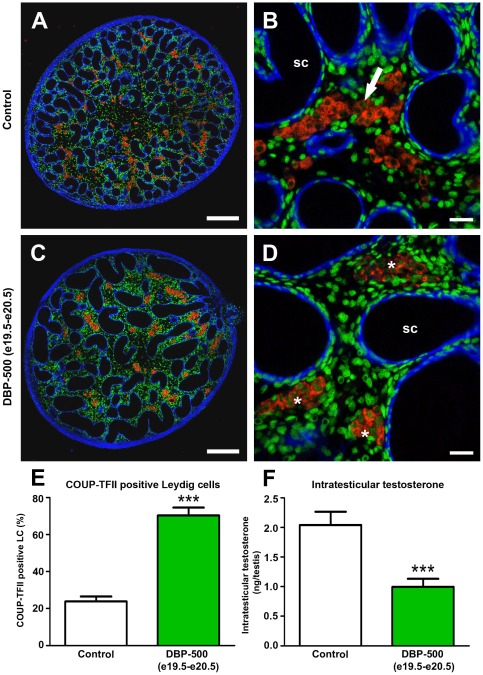
Altered COUP-TFII expression in fetal rat Leydig cells after *in utero* exposure to vehicle (control) or to 500 mg/kg/day dibutyl phthalate (DBP) from e19.5-e20.5 (late treatment window) and the relationship to intratesticular testosterone levels at e21.5. (A–D) Triple immunofluorescence for SMA (blue), 3β-HSD (red) and COUP-TFII (green) on testis sections from representative vehicle (control) and DBP-exposed animals on high resolution tiled images (A and C) and at higher power (B and D). Asterisks in panel D indicate Leydig cell aggregates that are predominantly immunopositive for COUP-TFII. SC = seminiferous cords. Scale bars in A and C = 200 µm, in B and D = 20 µm. (E) Quantification of the percentage of COUP-TFII positive fetal Leydig cells in animals from the treatment groups shown in panels A–D. Values are Means ± SEM for 8–10 animals per treatment group (minimum of 3 litters per group). ***p<0.001, in comparison with respective control. (F) Corresponding intratesticular testosterone levels for the treatment groups in panels A–D. Values are Means ± SEM for 18–20 animals per group (minimum of 3 litters per group). ***p<0.001, in comparison with respective control.

Several studies in the literature have shown that transfection of COUP-TFII into steroidogeneic cells results in antagonism of SF-1 action [18], [19], [20], [21], [22], [23], consistent with our observations. We therefore sought to obtain direct evidence for this in fetal rat LC by using a Lentivirus encoding COUP-TFII (Lv-COUP-TFII) to over-express COUP-TFII in isolated fetal testicular cells at e17.5 and thereby cause a reduction in *StAR*, *Cyp11a1* and *Cyp17a1* mRNA expression and testosterone production. Unfortunately, this *ex vivo* approach resulted in cell death after infection with LV-COUP-TFII (data not shown). There are also inherent problems with applying similar approaches to the *ex vivo* culture of fetal LC, which rapidly (∼48 h) lose their steroidogenic function after isolation [24]. Therefore, to provide more definitive evidence that nuclear COUP-TFII expression in fetal LC was associated *causally* with reduced ITT, DBP treatment was delayed until a time-point (e19.5–e20.5;  = late treatment window) when ∼80% of LC have normally switched off nuclear expression of COUP-TFII (Fig. 5). This ‘late window’ exposure to DBP-500 re-induced COUP-TFII expression in ∼70% of the fetal LC at e21.5 (Fig. 6D,E), while control (corn oil-exposed) animals only expressed COUP-TFII in a minority of fetal LC (Fig. 6B,E). Late window exposure to DBP-500 also resulted in a >50% reduction in ITT compared with controls (Fig. 6F).

The above results are consistent with cause and effect between DBP-induction or prolongation of COUP-TFII expression in fetal LC and suppression of ITT. We therefore investigated whether other treatments which have been shown to reduce fetal testosterone production in rats might target the same mechanism.

### Effects of exposure to DBP ± Dex on fetal LC nuclear expression of COUP-TFII and ITT

**Figure 7:**
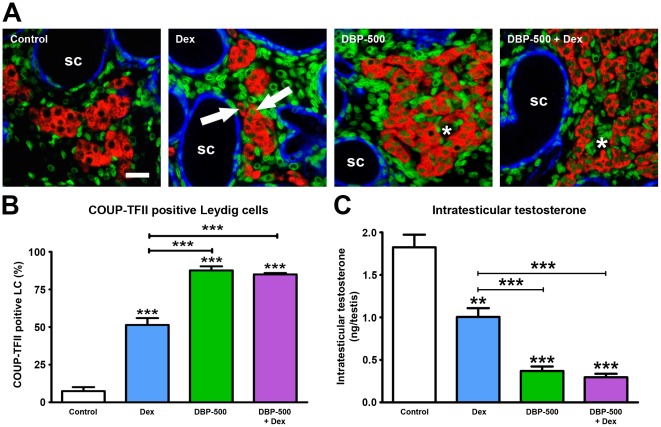
Altered COUP-TFII expression in fetal rat Leydig cells after *in utero* exposure to vehicle (control), to dexamethasone (Dex; 100 µg/kg/day) to dibutyl phthalate (DBP; 500 mg/kg/day) or combined DBP + Dex and the relationship to intratesticular testosterone levels at e21.5. (A) Triple immunofluorescence for SMA (blue), 3β-HSD (red) and COUP-TFII (green) on testis sections from representative vehicle (control) and DBP±Dex-exposed animals on higher power images. Note that exposure to Dex alone resulted in increased occurrence of COUP-TFII-immunopositive fetal Leydig cells (arrows) compared with controls and that combined exposure to DBP-500 + Dex or exposure to DBP-500 alone resulted in most Leydig cells being immunopositive for COUP-TFII (asterisks). SC = seminiferous cords. Scale bar = 20 µm. (B) Quantification of the percentage of COUP-TFII positive fetal Leydig cells in animals from the treatment groups shown in panel A. Values are Means ± SEM for 3–6 animals per treatment group (minimum of 3 litters per group). ***p<0.001, in comparison with respective control; other comparisons are indicated by capped lines. (C) Corresponding intratesticular testosterone levels for the treatment groups in panel A. Values are Means ± SEM for 19–22 animals per group (minimum of 3 litters per group). **p<0.01, ***p<0.001, in comparison with respective control; other comparisons are indicated by capped lines.

We have previously shown that *in utero* exposure to the synthetic glucocorticoid dexamethasone (Dex) amplifies the suppressive effects of DBP on fetal testis ITT and may thus induce more severe TDS disorders [3]. Dex exposure alone (e13.5–e20.5) increased the percentage of fetal LC expressing COUP-TFII at e21.5 to 50% compared with ∼8% in vehicle-exposed controls (Fig. 7A,B). In comparison, exposure to DBP-500 increased the proportion of COUP-TFII-positive LC to ∼85%, and co-exposure to both DBP-500 + Dex caused a similar magnitude of change (Fig. 7A,B). The DBP ± Dex-induced changes in COUP-TFII expression in LC again showed a striking inverse relationship to ITT (Fig. 7C) (r^2^ = 0.89, p<0.0001, linear regression analysis between left testis ITT and right testis percentage of COUP-TFII positive fetal LC). Exposure to DBP ± Dex reduced the mRNA expression of *Cyp11a1*, *StAR* and *Cyp17a1* (Fig. S3A–C), with the degree of suppression paralleling the percentage of fetal LC that expressed COUP-TFII in their nuclei (Fig. 7B). In contrast to the effects on *Cyp11a1*, *StAR* and *Cyp17a1*, DBP ± Dex exposure had no effect on the expression of another LC-specific gene *3β-HSD* (Fig. S3D). To further explore a possible link between COUP-TFII expression and suppression of SF-1-dependent genes, we investigated whether DBP ± Dex exposure had any effect on the expression of the SF-1 target gene *Amh* in Sertoli cells, as COUP-TFII is absent from Sertoli cells at all ages and treatments studied (Figs. 4,5,6,7). As shown in Figure S3E, *Amh* gene expression in Sertoli cells was unaffected by DBP ± Dex exposure. LHR-mediated drive to LC steroidogenesis may become progressively important in late gestation (beyond e18.5) in rats [1], but none of the treatments significantly altered *LHR* gene expression at e21.5 (Fig. S4A); we were unable to measure fetal LH levels due to lack of sufficient blood.

### Effects of fetal exposure of rats to DES on expression of COUP-TFII, ITT and SF-1 target gene expression

**Figure 8:**
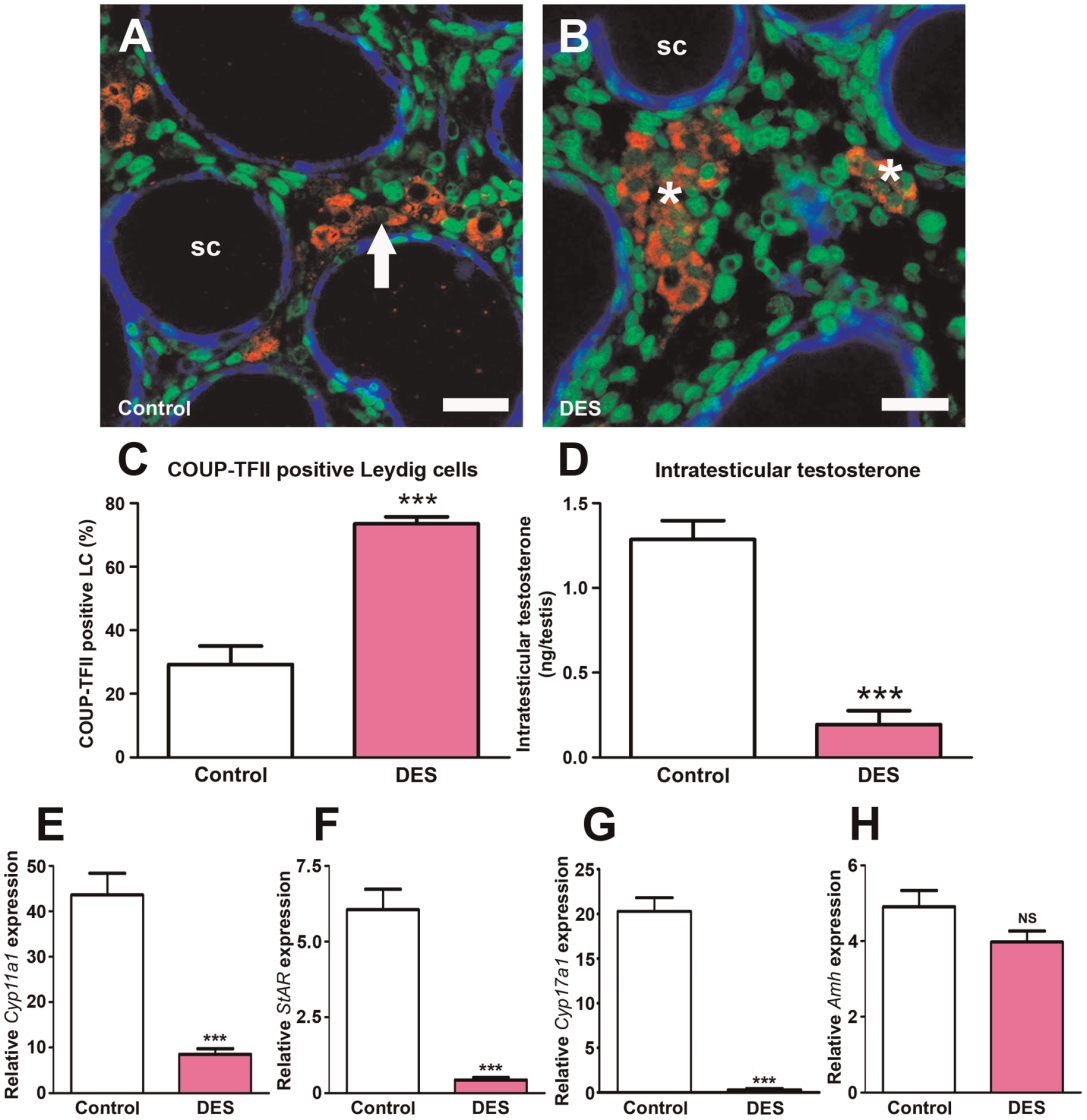
Effect of *in utero* exposure of rats to vehicle (control) or to diethylstilbestrol (DES 100 µg/kg on e13.5, e15.5, e17.5, e19.5 and e20.5) on COUP-TFII immunoexpression in fetal Leydig cells at e21.5. (A) Triple immunofluorescence for SMA (blue), 3β-HSD (red) and COUP-TFII (green) on testis sections from representative vehicle (control) and DES-exposed animals. Note that in controls occasional fetal Leydig cells are COUP-TFII-immunopositive (arrow) whereas exposure to DES resulted in a 3-fold increase in the % of COUP-TFII-immunopositive Leydig cells (asterisks). SC = seminiferous cords. Scale bar = 20 µm. (B) Quantification of the percentage of COUP-TFII positive fetal Leydig cells in animals from the treatment group shown in panel A. Values are Means ± SEM for 3–13 animals per treatment group (minimum of 3 litters per group). ***p<0.001, in comparison with respective control. (C) Corresponding intratesticular testosterone levels for the treatment groups in panel A. Values are Means ± SEM for 13–25 animals per group (minimum of 3 litters per group). ***p<0.001, in comparison with respective control. (E) cytochrome P450, family 11, subfamily a, polypeptide 1 (*Cyp11a1*), (F) Steroidogenic acute regulatory protein *(StAR*), (G) cytochrome P450, family 17, subfamily a, polypeptide 1 (*Cyp17a1*), (H) Anti-Müllerian hormone (*Amh*) gene expression in testes from control and DES-exposed males at e21.5. Values are Means ± SEM for 11–24 animals per group (minimum of 3 litters per group). ***p<0.001, in comparison with respective control. NS = not significant.

Previous research has shown that *in utero* exposure of rats to diethylstilbestrol (DES) results in reduced ITT [25]. Therefore, we investigated whether the DES-induced decrease in ITT was related to altered immunoexpression of COUP-TFII in the fetal LC at e21.5. *In utero* exposure to DES resulted in an increase in the percentage of COUP-TFII-positive LC when compared to controls (Figs. 8A–C), which again correlated inversely with ITT levels (Fig. 8D). Testes from DES-exposed males had significantly reduced expression of SF-1 target genes in fetal LC, *Cyp11a1* (Fig. 8E), *StAR* (Fig. 8F) and *Cyp17a1* (Fig. 8G), whereas the SF-1 target gene in Sertoli cells, *Amh*, was unaffected by DES treatment (Fig. 8H). The more pronounced suppression of both ITT and LC gene expression found after DES treatment, when compared with that found after DBP treatment (Fig. 2,3), is probably explained by the pronounced suppression of *LHR* expression found at e21.5 in DES-exposed animals (thus limiting LH-stimulation of the LC), a change not found after DBP exposure (Fig. S4).

### DBP exposure of mice does not alter COUP-TFII nuclear expression in fetal LC and has no effect on ITT, whereas exposure to DES does

**Figure 9:**
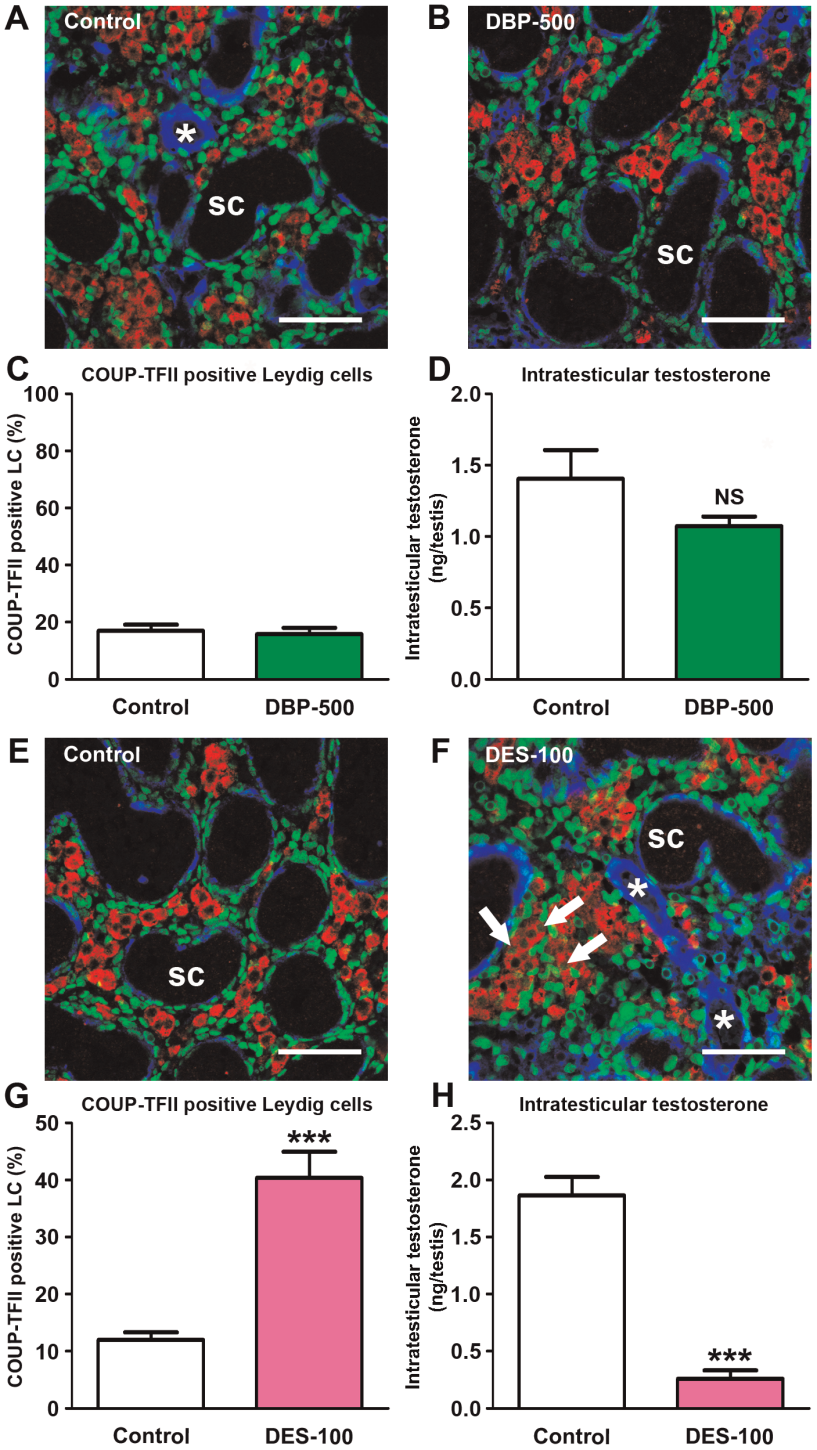
Effect of *in utero* exposure of mice to vehicle (control), dibutyl phthalate (DBP 500 mg/kg/day) or to diethylstilbestrol (DES 100 µg/kg on e11.5, e13.5, e15.5 and e17.5) on COUP-TFII immunoexpression in fetal Leydig cells at e18.5. (A–B, E–F) Triple immunofluorescence for SMA (blue), 3β-HSD (red) and COUP-TFII (green) on testis sections from representative vehicle (control; A, E), DBP-exposed (B) and DES-exposed (F) animals. Scale bars = 50 µm. Asterisks indicate blood vessels. Arrows in F indicate COUP-TFII-positive Leydig cells. (C–D) Quantification of the percentage of COUP-TFII positive fetal Leydig cells (C) and corresponding intratesticular testosterone levels (D) in control and DBP-exposed animals. Values are Means ± SEM for 7 animals per group (minimum of 3 litters per group). (G–H) Quantification of the percentage of COUP-TFII positive fetal Leydig cells (G) and corresponding intratesticular testosterone levels (H) in control and DES-exposed animals. Values are Means ± SEM for 6–9 animals per group (minimum of 3 litters per group). ***p<0.001, in comparison with respective control. NS = not significant.

In contrast to rats, gestational exposure of mice to DBP has no effect on ITT (Fig. 9D) or on LC steroidogenic enzyme expression [26], [27], a species difference that is unexplained. We investigated if a species-specific difference in the effect of DBP on nuclear expression of COUP-TFII in fetal LC might explain this. At e18.5 in mice (equivalent to e21.5 in rats) gestational exposure to DBP-500 (treatment since e11.5) did not alter the percentage of fetal LC nuclei that were immunopositive for COUP-TFII; the percentage remained low as in vehicle-exposed controls (Fig. 9A–C), in contrast to the results in DBP-exposed rats (Figs. 4B,5C). However, as previously reported [28], we found that exposure of pregnant mice to diethylstilbestrol (DES) resulted in a reduction in ITT in male fetuses. In mice, as in rats (Fig. 8), DES-exposure resulted in 40% of the fetal LC being positive for COUP-TFII compared to 12% in controls (Fig. 9E–G). This correlated inversely with ITT after DES-exposure which was reduced by 7-fold in DES-exposed animals when compared with controls (Fig. 9H).

### COUP-TFII expression in human fetal LC is down-regulated during development

**Figure 10:**
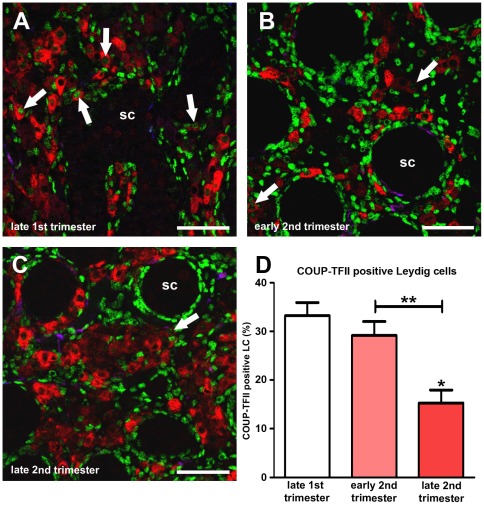
COUP-TFII expression in fetal Leydig cells in human fetal testis samples from late 1^st^ trimester (A), early 2^nd^ trimester (B) and late 2^nd^ trimester (C). Triple immunofluorescence for SMA (blue), 3β-HSD (red) and COUP-TFII (green) on human fetal testis sections. SC = seminiferous cords. Scale bars = 50 µm. (D) Quantification of the percentage of COUP-TFII positive fetal Leydig cells in samples shown in panels A–C. Values are Means ± SEM for 3–11 samples per treatment group. *p<0.05, **p<0.01, in comparison with respective control; other comparison is indicated by capped line.

To evaluate whether these findings in rodents are relevant in humans, we quantified fetal LC expression of nuclear COUP-TFII in testes from late 1^st^ trimester, early 2^nd^ trimester and late 2^nd^ trimester samples. This showed that the percentage of LC nuclei that were immunopositive for COUP-TFII declined over this developmental time period (Fig. 10), a trend similar to, although less pronounced, than that observed in control rat testes between e15.5–e21.5 (Fig. 5). Very early 1^st^ trimester samples, equivalent to e15.5 in the rat, were unavailable for study.

### Fetal LC from complete androgen receptor knockout (ARKO) mice are predominantly immunonegative for COUP-TFII expression

Since reduced ITT was found in every instance when there was abnormal maintenance/induction of COUP-TFII expression in fetal rodent LC, we considered whether the former could be driving the latter. This reverse causation seems unlikely, since in complete androgen receptor knockout (ARKO) mice the fetal LC at e18.5 are predominantly immunonegative for COUP-TFII (Fig. S5).

## Discussion

The present study has identified a novel mechanism that could potentially play a key role in up-regulating testosterone production by rat fetal LC during and after the critical masculinization programming window (MPW) [1], [2]. The mechanism we propose is a progressive age-related reduction in expression of COUP-TFII in fetal LC, which effectively removes repression by a competitor with SF-1 for binding to overlapping sites in the promoter region of steroidogenic enzyme genes. The present results show that this mechanism is perturbed by exposure to three separate factors in the rat, some of which may be relevant to the human and other species. This mechanism could also partly account for the previously unexplained ‘paracrine’ regulation of fetal LC steroidogenesis during the MPW in rats and humans [1]. Our proposal would add another tier of evidence for the COUP-TF family being active repressors of key elements in the male reproductive system, ranging from luteinizing hormone expression (*LHβ*; [29]) through LH receptor expression (*LHR*; [30], [31], [32]) to fetal LC function (this study). Our results also identify, for the first time, a primary mechanism by which phthalates, such as DBP, inhibit steroidogenesis by fetal LC in the rat (but not in the mouse). Our studies show that the COUP-TFII mechanism is present in human fetal LC, but whether it plays a role analogous to that which we propose in the rat will depend on further studies.

Our results provide convincing time course and dose-response evidence that exposure to DBP, DES and to a lesser extent dexamethasone, prevent the normal time-dependent down-regulation of nuclear COUP-TFII that occurs in fetal LC in the rat and which is associated temporally with expansion of LC cytoplasmic volume (which harbors the steroidogenic organelles) and increase in ITT. The temporal changes fit with the demonstration that DBP-induced down-regulation of SF-1-dependent LC-specific genes first emerges at e17.5 [12], consistent with this being the earliest age at which COUP-TFII expression in LC in control animals is down-regulated, a change prevented by DBP-treatment. We show that treatment-induced changes in LC nuclear COUP-TFII expression are, in all instances, associated with inverse changes in ITT and with altered expression of SF-1-dependent LC-specific genes that have shared/overlapping SF-1 and COUP-TFII response elements in their promoter regions (Table 1). In contrast, expression of the LC steroidogenic gene (*3β-HSD*) that does not have an overlapping SF-1 and COUP-TFII response element in its promoter, was unaffected by DBP exposure, as was expression of *Amh* in Sertoli cells; in the latter case, there are separate SF-1 and COUP-TFII response elements in the promoter of *Amh* (Table 1), but in any case COUP-TFII was never expressed in Sertoli cells in our studies. Therefore, we show a robust association between the LC-specific expression of COUP-TFII, reduced ITT and the down-regulation of steroidogenic genes that have overlapping SF-1 and COUP-TFII response elements.

Our identification of altered COUP-TFII expression in fetal rat LC as a mechanism underlying suppression of ITT resulting from experimental treatments (DBP and/or Dex or DES) is based on showing a consistent inverse *association* between the percentage of fetal LC expressing COUP-TFII in their nuclei and ITT levels. This association does not in itself prove ‘cause and effect’. The ideal way of proving this would be to over-express COUP-TFII in fetal LC and show this reduces testosterone production. Such studies have been done with adult-derived bovine steroidogenic cells via transfection and shown to result in reduced steroidogenesis and expression of *StAR* and *Cyp17a1* [18], [19], [20], as found in the present association studies. Numerous studies have shown that the mechanism underlying such effects involves competition between COUP-TFII and SF-1 for binding to an overlapping response element in the promoter region of genes encoding steroidogenic enzymes [18], [19], [20], [21], [23], [30], [31], [32], [33], [34], [35], [36], [37], [38], as proposed for the present studies in fetal rat LC. Unfortunately, our studies using viral transfection of *ex vivo* cultured rat fetal LC with COUP-TFII resulted in cell death (unpublished data), and there are also inherent problems with the culture of fetal LC, which rapidly lose their steroidogenic function [24]. Therefore, this direct approach was not an option for us. We therefore decided on two alternative approaches to provide stronger evidence for causation, one involving re-induction of COUP-TFII in rat fetal LC (by DBP treatment) after its age-related loss, and the second involving parallel studies in the mouse in which DBP had been shown by others to be incapable of suppressing steroidogenesis and the expression of SF-1-dependent genes [26], [27], [39].

For the first approach, we exposed pregnant rats to DBP at a time in gestation (from e19.5–e20.5) when COUP-TFII had already switched off in the majority of fetal LC. This ‘late window’ DBP treatment resulted in re-induction of COUP-TFII expression in most of the fetal LC and an associated reduction in ITT at e21.5, consistent with our mechanistic proposal. In our mouse studies we confirmed that DBP exposure had no effect on ITT, nor was there induction/maintenance of COUP-TFII expression in fetal LC. However, exposure of pregnant mice to DES, rather than DBP, did result in profound suppression of ITT and a corresponding increase in the percentage of fetal LC expressing COUP-TFII, a change that paralleled that found for DES in the rat. The degree of suppression of ITT induced by DES was notably larger than that induced by DBP (in the rat), a difference probably explained by a parallel reduction in LH drive to the LC due to reduced *LHR* expression. This raises the possibility that LH secretion, which is initiated at ∼e18.5 in the rat and increases progressively thereafter [1], might be involved in switching off the expression of COUP-TFII in fetal LC and that DBP causes its steroidogenic effects by suppressing LH. As we were unable to measure fetal LH in blood, we could not test this possibility directly, but existing data suggests it is an unlikely explanation for our findings. First, it would fail to explain why the effects of DBP on ITT and steroidogenic enzyme expression in rats are first detectable at e17.5 (this study and [12]), an age prior to the production of LH in the rat [1]. Second, *in vitro* studies using rat fetal testis cultures show that phthalate metabolites inhibit testosterone production regardless of the absence or presence of LH in the culture media [40]. Nevertheless, even if DBP did suppress LH, it would appear that this suppression then results locally in a failure of COUP-TFII to switch off normally in fetal LC, which would still represent the causal mechanism within the LC. Moreover, if DBP exposure should inhibit fetal LH secretion, it is likely to involve a similar mechanism to that which we propose for the fetal LC, as COUP-TFII has been shown to competitively antagonize SF-1-induced *LHβ* expression in the adult pituitary gland [29].

We considered reverse causation as an alternative explanation for our findings, namely that because reduced ITT was found in every instance in which there was abnormal maintenance/induction of COUP-TFII expression in fetal LC, then the former could be driving the latter. We consider this unlikely, because in complete androgen receptor knockout (ARKO) mice the fetal LC at e18.5 are predominantly immunonegative for COUP-TFII and, second, in the rat most fetal LC do not express the androgen receptor and are thus not directly androgen-responsive [41].

Our analyses of COUP-TFII expression in fetal LC used confocal microscopy and identification of LC by cytoplasmic staining for 3β-HSD. We were able to do this because, unlike the other SF-1-regulated LC steroidogenic genes, expression of *3β-HSD* was unaffected in any of our treatment groups. Use of high resolution tiled images of complete fetal testis cross-sections allowed us to identify fetal LC unequivocally and to specifically assess the presence or absence of COUP-TFII expression in individual LC. Since COUP-TFII is abundantly expressed in other cell types in the fetal testis, especially in non-Leydig interstitial cells, whole testis measurements such as the analysis of total testicular *COUP-TFII* mRNA expression would not be meaningful, and, indeed, we found no effect of DBP-exposure on overall *COUP-TFII* mRNA expression in the fetal rat testis. We saw no evidence for altered COUP-TFII expression in the non-Leydig interstitial cells in the fetal testis, and these cells did not affect our analyses because these were focused only on identifiable fetal LC (ie cells expressing 3β-HSD in their cytoplasm). We chose an antibody dilution for detection of COUP-TFII immunoexpression that discriminated between immunonegative LC in controls and immunopositive LC in DBP-exposed animals. In reality, we think it likely that this distinction represents profound down-regulation, rather than complete absence, of COUP-TFII immunoexpression in the nuclei of late gestation fetal LC in controls, based on titration studies with the COUP-TFII antibody (Fig. S6).

Based on the age-related change in COUP-TFII immunoexpression in fetal human LC in the present studies, the mechanism which we propose for COUP-TFII in the rat may apply to the human, but more detailed studies are needed to support this possibility. This does not imply that each of the treatment effects shown to affect this mechanism in the rat will apply to the human, as our preliminary data is that in the human, as in mice, DBP neither affects steroidogenesis [42] nor COUP-TFII expression (our unpublished data), at least in a xenograft model system. Nevertheless, as we show that three separate factors can maintain/increase nuclear expression of COUP-TFII in fetal rat LC with associated decreases in fetal ITT, it suggests that the COUP-TFII mechanism is potentially vulnerable to a wider range of factors.

In conclusion, our results all point strongly towards COUP-TFII expression being a key (negative) regulator of steroidogenesis within fetal LC during and after the critical period for masculinization in the rat, and potentially in the human. Thus, lifting of steroidogenic repression by COUP-TFII, rather than direct stimulation of steroidogenesis by paracrine factors, could be the primary LH-independent mechanism responsible for increasing testosterone production to induce masculinization. Perturbation of this novel pathway is clearly linked via our DBP studies in the rat to downstream TDS disorders. We show that this pathway can be impacted by factors other than DBP, for example via the stress hormone axis (glucocorticoids) and by estrogens. We consider it likely that other factors (eg other environmental chemicals) also target this pathway. The present findings suggest new pathways by which lifestyle factors in combination with environmental chemicals could exert adverse effects and lead to TDS disorders.

## Methods

### Animals and treatments

Wistar rats and C57BL/6J mice were maintained according to UK Home Office guidelines (which also involves an ethical approval step) and were fed a soy-free breeding diet (RM3(E) soya free; SDS, Dundee, Scotland). Housing conditions were carefully controlled (lights on at 0700, off at 1900 h, temperature 19–21 C, GOLD shavings and LITASPEN standard bedding (SPPS, Argenteuil, France)). Time-mated female rats were subjected to the daily treatments described below. Depending on the age of termination, treatments were administered from embryonic day (e) 13.5–e14.5 (termination e15.5), e13.5–e16.5 (termination e17.5), e13.5–e18.5 (termination e19.5), e13.5–e20.5 (termination e21.5) or e13.5–e21.5 (termination in adulthood) between 0900 and 1030 h. In a different set of experiments treatments were administered from e19.5–e20.5 (termination e21.5; late treatment window). The doses of dibutyl phthalate (DBP), dexamethasone (Dex) and diethylstilbestrol (DES) were based on previous studies [3], [9], [25], [43]. The DBP was 99% pure according to the supplier. Rat treatment groups were as follows:

1. DBP (Sigma-Aldrich Co. Ltd., Dorset, UK) at a dose of either 20, 100 or 500 mg/kg administered by oral gavage in 1 ml/kg corn oil, plus daily subcutaneous injection of 1 ml/kg saline (vehicle control for Dex).
2. Dex (Sigma-Aldrich) at a dose of 100 µg/kg/day by subcutaneous injection in 1 ml/kg saline plus 1 ml/kg corn oil by oral gavage (vehicle control for DBP).
3. A combination of DBP (500 mg/kg by oral gavage) plus Dex (100 µg/kg/day subcutaneously).
4. DES (Sigma-Aldrich) at a dose of 100 µg/kg in 1 ml/kg corn oil by subcutaneous injection on e13.5, e15.5, e17.5, e19.5 and e20.5.
5. Control (1 ml/kg corn oil by gavage and 1 ml/kg saline by subcutaneous injection).

Additionally, pregnant female mice were treated with DBP (500 mg/kg by oral gavage from e11.5 to e17.5) or DES (100 µg/kg by subcutaneous injection on e13.5, e15.5 and e17.5) and were terminated on e18.5. Androgen receptor knockout (ARKO) mice were generated as described previously [44], and were terminated at e18.5.

### Tissue recovery, processing and adult rat phenotyping

To acquire fetal samples, rat dams were killed by inhalation of CO_2_ followed by cervical dislocation at e15.5, e17.5, e19.5, e21.5 or adulthood and mouse dams at e18.5. Fetuses were removed, decapitated and placed in ice cold phosphate buffered solution (PBS; Sigma-Aldrich). Testes were microdissected, and fixed in Bouin's fixative for 1 hour at room temperature or snap frozen and stored at −70°C for gene expression analysis or determination of intratesticular testosterone (ITT) by homogenizing the testis and measuring its total testosterone content using a radioimmunoassay as described previously [9]. The limit of detection of the testosterone assay was 40 pg and the intra- and inter-assay CVs were <9% and <14%, respectively. Bouin's-fixed tissues were processed and embedded in paraffin wax, and 5-µm sections were used for subsequent experiments. Adult rats exposed to 500 mg/kg DBP or vehicle control were subjected to a thorough inspection to determine the normality of the penis and testicular descent as described previously [3]. In addition the testes of these adult rats were dissected and weighed.

### Human fetal testis samples

First- and second-trimester testes were obtained after medical termination of pregnancy for social reasons as described previously [45]. Written maternal consent was obtained, and the study was approved by the Lothian Research Ethics Committee. Gestation was determined by ultrasound scan and subsequent direct measurement of foot length. The sex of first-trimester testes was confirmed by PCR for the male-specific gene *SRY*. Testes were removed and fixed in Bouin's fixative for 2 hours before processing into paraffin using standard methods. A total of 24 fetal specimens were used in this study: 3 late 1^st^ trimester samples (<12 weeks), 10 early 2^nd^ trimester samples (12–17 weeks) and 11 late 2^nd^ trimester samples (18–20 weeks).

### Determination of Leydig (3β-hydroxysteroid dehydrogenase-immunopositive) cell number, nuclear volume and cytoplasmic volume per testis

Testicular sections from 4–8 animals per age/treatment group were immunostained for 3β-hydroxysteroid dehydrogenase (3β-HSD) as described previously [46] and counterstained with hematoxylin. The volume of Leydig (3β-HSD-positive) cells per testis was determined using stereological methods similar to those described previously [47]. Briefly, three (non-serial) sections per animal were analyzed using a Zeiss Axio-Imager microscope (Carl Zeiss Ltd., Welwyn Garden City, UK) fitted with a Hitachi HV-C20 camera (Hitachi Denshi Europe, Leeds, UK) and a Prior automatic stage (Prior Scientific Instruments Ltd., Cambridge, UK). Image-Pro 6.2 with Stereologer plug-in software (MagWorldwide, Wokingham, UK) was used to select random fields and to place a counting grid over the tissue. The total number of fields counted per animal (∼65–95 fields) was dependent on obtaining a percentage SE value of <5%. Points falling over 3β-HSD-positive cytoplasm, or over the nuclei of cells with 3β-HSD-positive cytoplasm, were scored separately, and both were independently expressed as relative volumes per testis. These data were converted to absolute volume per testis by multiplying by testis weight (equivalent to volume). Data for LC nuclei were then converted to cell number per testis after determination of mean LC nuclear diameter and volume (∼100 nuclei per animal) using the Stereologer software nucleator function. Average LC cytoplasmic volume was calculated by dividing total LC cytoplasmic volume per testis by the number of LC.

### Immunofluorescence for Smooth Muscle Actin, 3β-HSD and COUP-TFII

In order to delineate the seminiferous cord compartment from the interstitial compartment and to distinguish COUP-TFII positive fetal LC from other COUP-TFII positive interstitial and peritubular myoid cells, specific antibodies were used for the co-immunolocalization of α-smooth muscle actin (α-SMA; clone 1A4, Sigma-Aldrich), 3β-HSD (for rat and mouse: clone P-18, Santa Cruz Biotechnology, Inc., CA, USA; for human: the antibody was a kind gift of professor Ian Mason) and COUP-TFII (clone H7147, R&D Systems, MN, USA). All washes between incubation steps were in TBS (3×5 min) and all incubations were carried out in a humidity box (Fisher Scientific, UK). Sections were dewaxed and rehydrated, followed by a peroxidase block in 3% (v/v) H_2_O_2_ in methanol for 30 min. Next, the sections were blocked in normal rabbit serum (NRS; Biosera, Ringmer, UK) diluted 1∶5 in TBS containing 5% (w/v) BSA (NRS/TBS/BSA), followed by incubation with anti-SMA antibody diluted 1∶10,000 in NRS/TBS/BSA for 1 hour at room temperature (RT). Sections were then incubated with peroxidase-conjugated rabbit anti-mouse secondary antibody (RAMP; DAKO Corp., Cambridge, UK), diluted 1∶200 in NRS/TBS/BSA for 30 minutes at RT, followed by incubation with Tyr-Cy5 (Perkin Elmer-TSA-Plus Cyanine5 System; Perkin Elmer Life Sciences, Boston, MA, USA) according to the manufacturer's instructions. Sections were then subjected to antigen retrieval by boiling in a pressure cooker in 0.01 mol/l citrate buffer (pH 6.0) for 5 min and left to cool for 20 minutes, followed by another block in NRS/TBS/BSA and overnight incubation at 4°C with anti-3β-HSD antibody diluted 1∶8,000 in NRS/TBS/BSA. Slides were then incubated with peroxidise-conjugated rabbit anti-goat secondary antibody (Sigma-Aldrich) diluted 1∶200 in NRS/TBS/BSA for 30 minutes at RT, followed by incubation with Tyr-Cy3 (Perkin Elmer-TSA-Plus Cyanine3 System; Perkin Elmer Life Sciences) according to the manufacturer's instructions. Sections were again blocked against peroxidase in 3% (v/v) H_2_O_2_ in TBS plus 0.01% (v/v) Tween-20 (Sigma-Aldrich) for 20 min followed by blocking in NRS/TBS/BSA and overnight incubation at 4°C with anti-COUP-TFII antibody diluted 1∶1,000 in NRS/TBS/BSA. Finally, on the third day, sections were incubated with RAMP diluted 1∶200 in NRS/TBS/BSA for 30 minutes at RT, and followed by incubation with Tyr-fl (Perkin Elmer-TSA-Plus Fluorescein System; Perkin Elmer Life Sciences) according to the manufacturer's instructions. Fluorescent images were captured using a Zeiss LSM 710 Axio Observer Z1 confocal laser microscope (Carl Zeiss Ltd.). All images were compiled using Photoshop 9.0 (Adobe Systems Inc.).

### Quantification of COUP-TFII positive fetal LC

Preliminary studies showed that, at e21.5, when DBP (500 mg/kg/day) exposure reduces ITT, this was associated with a high percentage of fetal LC expressing COUP-TFII in their nuclei, whereas in controls most LC nuclei were negative for COUP-TFII (Fig. 4,5). To validate this observation, COUP-TFII immunoexpression in LC nuclei in control and DBP-exposed animals was evaluated using serial dilutions of COUP-TFII antibody. This showed unequivocally that the level of COUP-TFII immunoexpression was considerably higher in DBP-exposed animals than in controls (Fig. S6). Based on these studies, an antibody dilution of 1∶1000 was chosen for the remaining studies, as this discriminated LC nuclear COUP-TFII immunoexpression clearly between control and DBP-exposed animals at a dose of DBP that was associated with induction of TDS disorders (Fig. 1). High resolution tiled confocal scanning laser microscopy images of complete testis cross sections co-stained for SMA, 3β-HSD and COUP-TFII were generated and used for determining the proportion of fetal LC which stained positively for COUP-TFII. At least 5 different testes per age and treatment group were used for counting. Briefly, exported images were opened using Image-Pro 6.2 software (MagWorldwide) and all LC in the image were counted (range in numbers = 25–1693; median 472 per testis cross-section) and scored negative or positive for nuclear COUP-TFII staining.

### Gene expression analysis at e21.5

For quantitative analysis of gene expression by RT-PCR, total RNA was extracted from e21.5 testis samples from the different treatment groups (controls, Dex-100 µg/kg, DBP-500 mg/kg, Dex-100 µg/kg + DBP-500 mg/kg, DES-100 µg/kg) using the RNeasy Micro Kit with on-column DNase digestion (Qiagen, UK). Random hexamer primed cDNA was prepared using the Applied Biosystems Taqman^TM^ RT kit (Applied Biosystems, CA). Quantitative real time PCR (qRT-PCR) was performed on the ABI Prism Sequence Detection System (Applied Biosystems). Expression of rat *StAR*, *Cyp11a1*, *Cyp17a1*, *3β-HSD*, *Amh*, *LHR* and *COUP-TFII* mRNA was determined using the Roche Universal Probe Library (*StAR* forward primer: 5′-TCACGTGGCTGCTCAGTATT-3′, reverse primer: 5′-GGGTCTGTGATAAGACTTGGTTG-3′, probe number 83 Cat no. 04689062001; *Cyp11a1* forward primer: 5′-TATTCCGCTTTGCCTTTGAG-3′, reverse primer 5′-CACGATCTCCTCCAACATCC-3′, probe number 9 Cat no. 04685075001; *Cyp17a1* forward primer: 5′-CATCCCCCACAAGGCTAAC-3′, reverse primer: 5′-TGTGTCCTTGGGGACAGTAAA-3′, probe number 67 Cat no. 04688660001; *Amh* forward primer: 5′-CTGGACACCGTGCCTTTC-3′, reverse primer: 5′-CACTGTGTGGCAGGTCCTC-3′, probe number 26 Cat no. 04687574001; *3β-HSD* forward primer: 5′-GACCAGAAACCAAGGAGGAA-3′, reverse primer: 5′-CTGGCACGCTCTCCTCAG-3′, probe number 105 Cat no. 04692241001; *LHR* forward primer: 5′-CTGGAGAAGATGCACAGTGG-3′, reverse primer 5′-CTGCAATTTGGTGGAAGAAATA-3′, probe number 107 Cat no. 04692268001; *COUP-TFII* forward primer: 5′-CGGAGGAACCTGAGCTACAC-3′, reverse primer 5′-CCACTTTGAGGCACTTTTTGA-3′, probe number 123 Cat no. 04693574001; Roche Applied Sciences, Burgess Hill, UK). The expression level of each gene was corrected using a ribosomal 18S internal control (Applied Biosystems Cat no. 4308329). All samples were performed in triplicate and a relative comparison was made to adult testis control cDNA. For each treatment group, at least fifteen e21.5 rat fetuses from 5 litters were analyzed.

### Immunohistochemistry for Cyp11a1, 3β-HSD and Amh

Specific protein expression of Cyp11a1, 3β-HSD and Amh were detected by immunohistochemistry on e21.5 testis sections isolated from control and DBP-exposed animals, using standard methods that have been detailed previously [12], [48]. The primary antibodies and their dilutions used in the present studies were as follows: rabbit anti-Cyp11a1 (1∶200; Chemicon International Inc., Temecula, CA, USA), goat anti-3β-HSD (1∶800; Santa Cruz Biotechnology, Santa Cruz, CA, USA), goat anti-Amh (1∶30; Santa Cruz Biotechnology, Santa Cruz, CA, USA).

### Statistical analysis

Data were analysed using GraphPad Prism version 5 (Graph Pad Software Inc., San Diego, CA) and one-way Analysis of Variance followed by the Bonferroni post-test, or student *t*-test when control and treated groups at a particular age were compared. The Fisher's exact test was used for comparing the incidence of cryptorchidism and hypospadias in vehicle- and DBP-exposed treatment groups. Data for fetal ITT and mRNA levels were log transformed prior to analysis to normalize variances.

## Supporting Information

Figure S1 **Effect of** ***in utero*** **exposure of rats to vehicle (control), or dibutyl phthalate (DBP 500 mg/kg/day) on the mRNA expression of** ***COUP-TFII*** **in the fetal testis at e21.5.** Values are Means ± SEM for 11–14 animals per group (minimum of 3 litters per group).
(TIF)

Figure S2 **COUP-TFII immunoexpression in e21.5 control testis (A) and corresponding image in (B) showing DAPI nuclear counterstain.** Arrows indicate examples of nuclear COUP-TFII/DAPI staining which has a “cytoplasmic” appearance, but in fact is all within the nucleus as indicated by DAPI staining. Scale bar = 20 µm.
(TIF)

Figure S3 **Effect of** ***in utero*** **exposure of rats to vehicle (control), Dexamethasone (Dex 100 µg/kg/day), dibutyl phthalate (DBP 500 mg/kg/day) or a combination of DBP-500 + Dex on steroidogenic enzyme and anti-Müllerian hormone gene expression in testes at e21.5.** (A) *Cyp11a1*, (B) *StAR*, (C) *Cyp17a1*, (D) *3β-HSD*, and (E) *Amh*. Note the lack of effect of treatments on expression of *3β-HSD* and *Amh.* Values are Means ± SEM for 19–22 animals per group (minimum of 5 litters per group). *p<0.05, **p<0.01, ***p<0.001, in comparison with respective control.
(TIF)

Figure S4 **Effect of** ***in utero*** **exposure of rats to (A) vehicle (control), dexamethasone (Dex 100 µg/kg/day), dibutyl phthalate (DBP 500 mg/kg/day) or a combination of DBP-500 + Dex or (B) diethylstilbestrol (DES 100 µg/kg) on luteinizing hormone receptor (** ***LHR*** **) gene expression in testes at e21.5.** Values are Means ± SEM for 11–24 animals per group (minimum of 3 litters per group). ***p<0.001, in comparison with respective control.
(TIF)

Figure S5 **COUP-TFII immunoexpression in fetal LC in control and complete androgen receptor knockout (ARKO) mice at e18.5.** Representative images of control and ARKO mice (n = 4) demonstrating that COUP-TFII is only rarely expressed (arrows) in ARKO fetal LC as in wild-type controls. Scale bar = 20 µm.
(TIF)

Figure S6 **Serial dilution of COUP-TFII antibody.** Triple immunofluorescence for SMA (blue), 3β-HSD (red) and COUP-TFII (green) on fetal testis sections from vehicle (control) and DBP-exposed (500 mg/kg/day) e21.5 animals. Note that in control sections most Leydig cells are COUP-TFII-immunopositive at low antibody dilutions (1∶250–1∶500) whereas only a minority is at lower antibody dilutions. In contrast, in sections from DBP-exposed animals, most Leydig cells are COUP-TFII-immunopositive at all antibody dilutions. SC = seminiferous cords. A wider range of antibody dilutions were run than is shown. Scale bar = 20 µm.
(TIF)
