# Supplementary figures and images for: Proposed Role for COUP-TFII in Regulating Fetal Leydig Cell Steroidogenesis, Perturbation of Which Leads to Masculinization Disorders in Rodents
Source: PLoS One. 2012 May 17;7(5):e37064. doi: 10.1371/journal.pone.0037064 (PMC3355148; doi:10.1371/journal.pone.0037064)

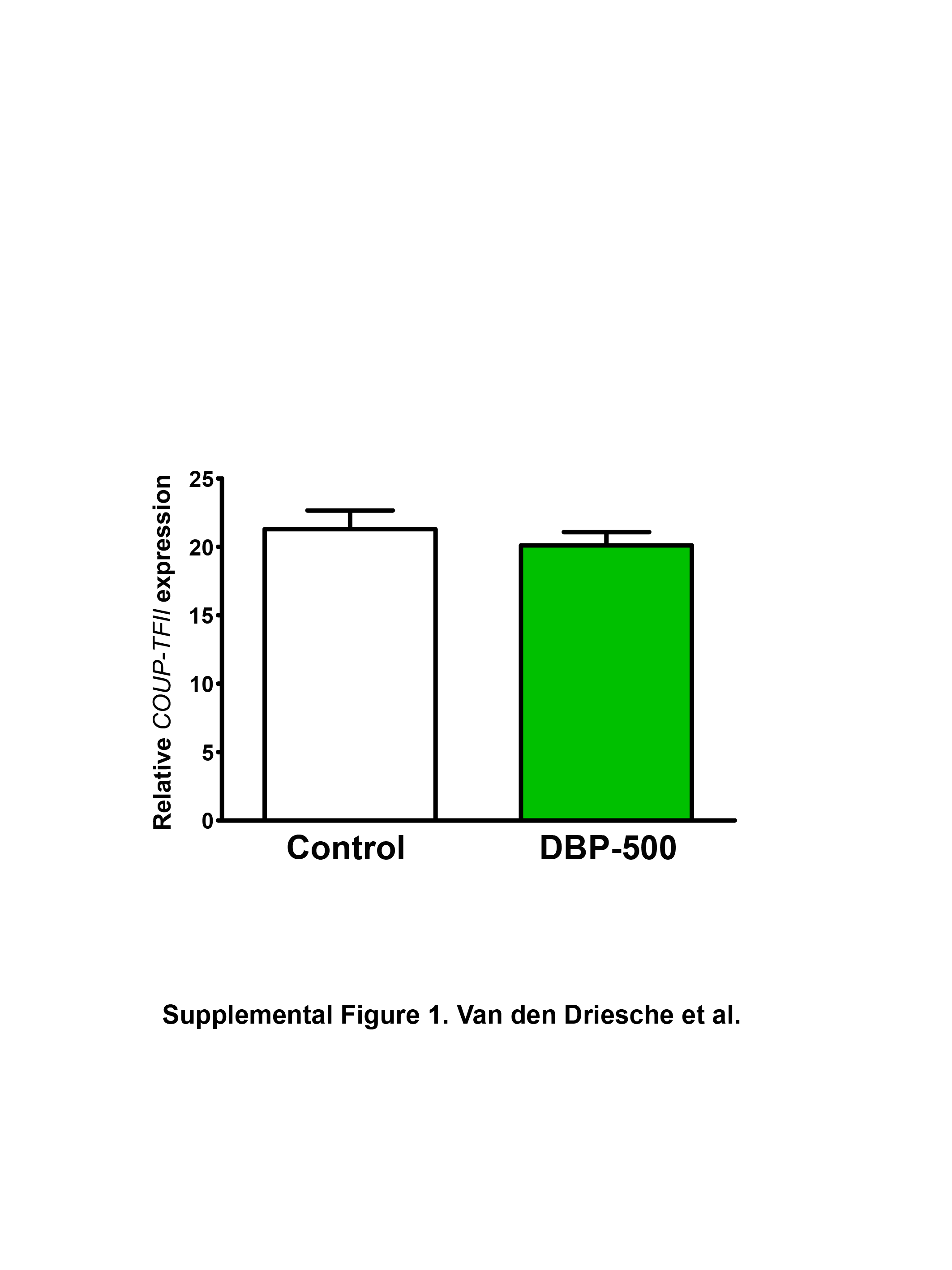

Supplement: Figure S1 — Effect of in utero exposure of rats to vehicle (control), or dibutyl phthalate (DBP 500 mg/kg/day) on the mRNA expression of COUP-TFII in the fetal testis at e21.5. Values are Means ± SEM for 11–14 animals per group (minimum of 3 litters per group). (TIF) [file pone.0037064.s001.tif]

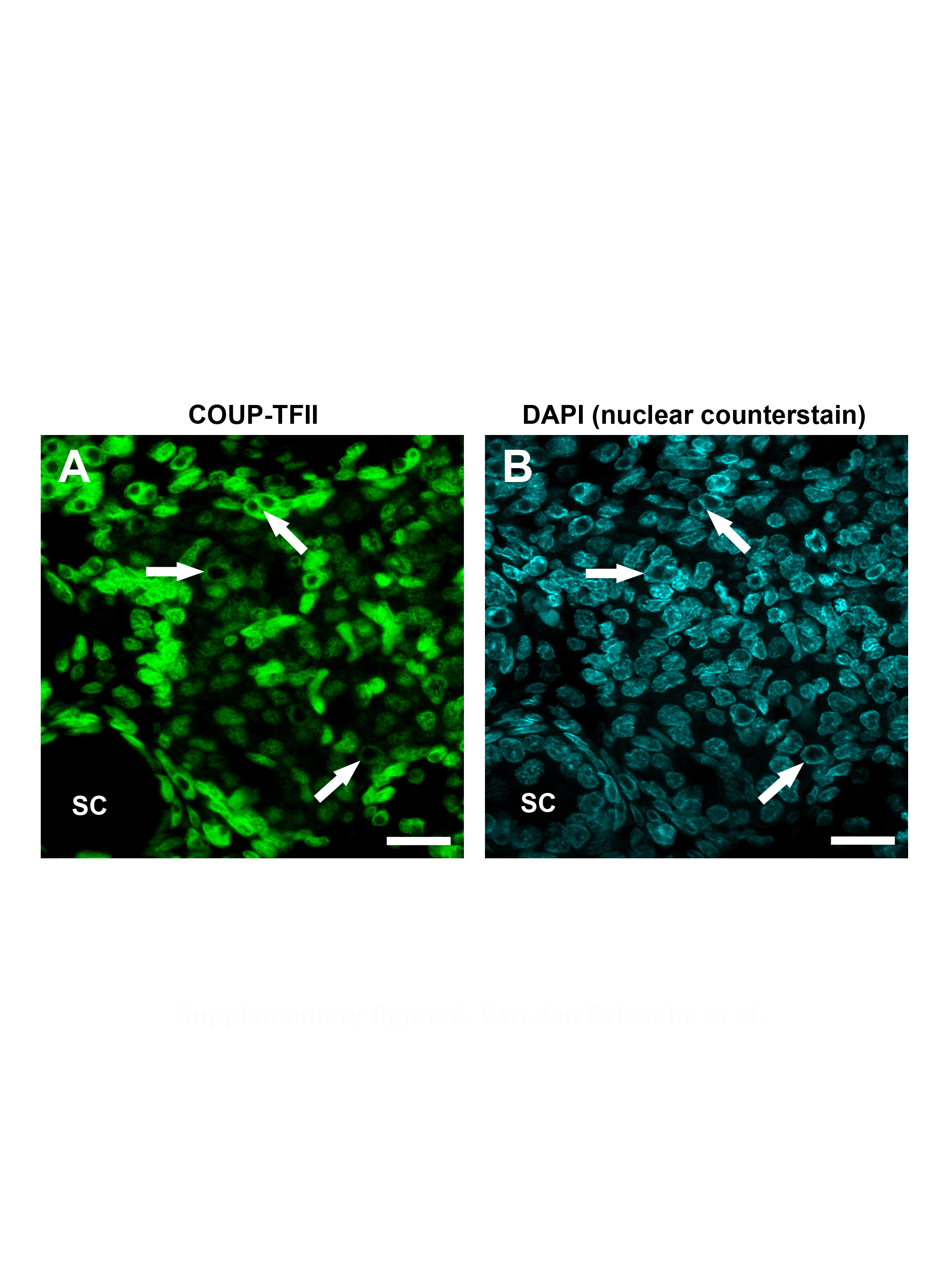

Supplement: Figure S2 — COUP-TFII immunoexpression in e21.5 control testis (A) and corresponding image in (B) showing DAPI nuclear counterstain. Arrows indicate examples of nuclear COUP-TFII/DAPI staining which has a “cytoplasmic” appearance, but in fact is all within the nucleus as indicated by DAPI staining. Scale bar = 20 µm. (TIF) [file pone.0037064.s002.tif]

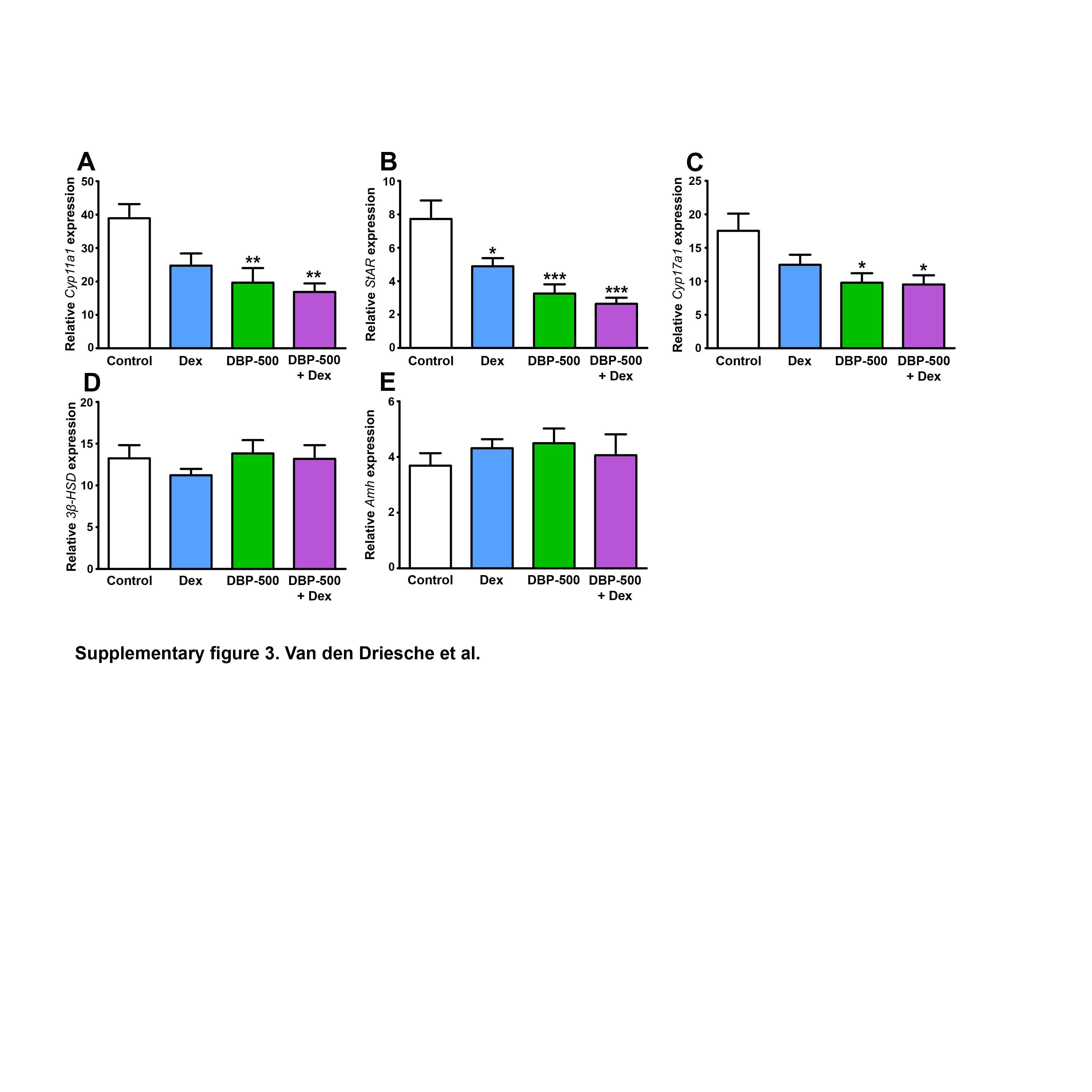

Supplement: Figure S3 — Effect of in utero exposure of rats to vehicle (control), Dexamethasone (Dex 100 µg/kg/day), dibutyl phthalate (DBP 500 mg/kg/day) or a combination of DBP-500 + Dex on steroidogenic enzyme and anti-Müllerian hormone gene expression in testes at e21.5. (A) Cyp11a1, (B) StAR, (C) Cyp17a1, (D) 3β-HSD, and (E) Amh. Note the lack of effect of treatments on expression of 3β-HSD and Amh. Values are Means ± SEM for 19–22 animals per group (minimum of 5 litters per group). *p<0.05, **p<0.01, ***p<0.001, in comparison with respective control. (TIF) [file pone.0037064.s003.tif]

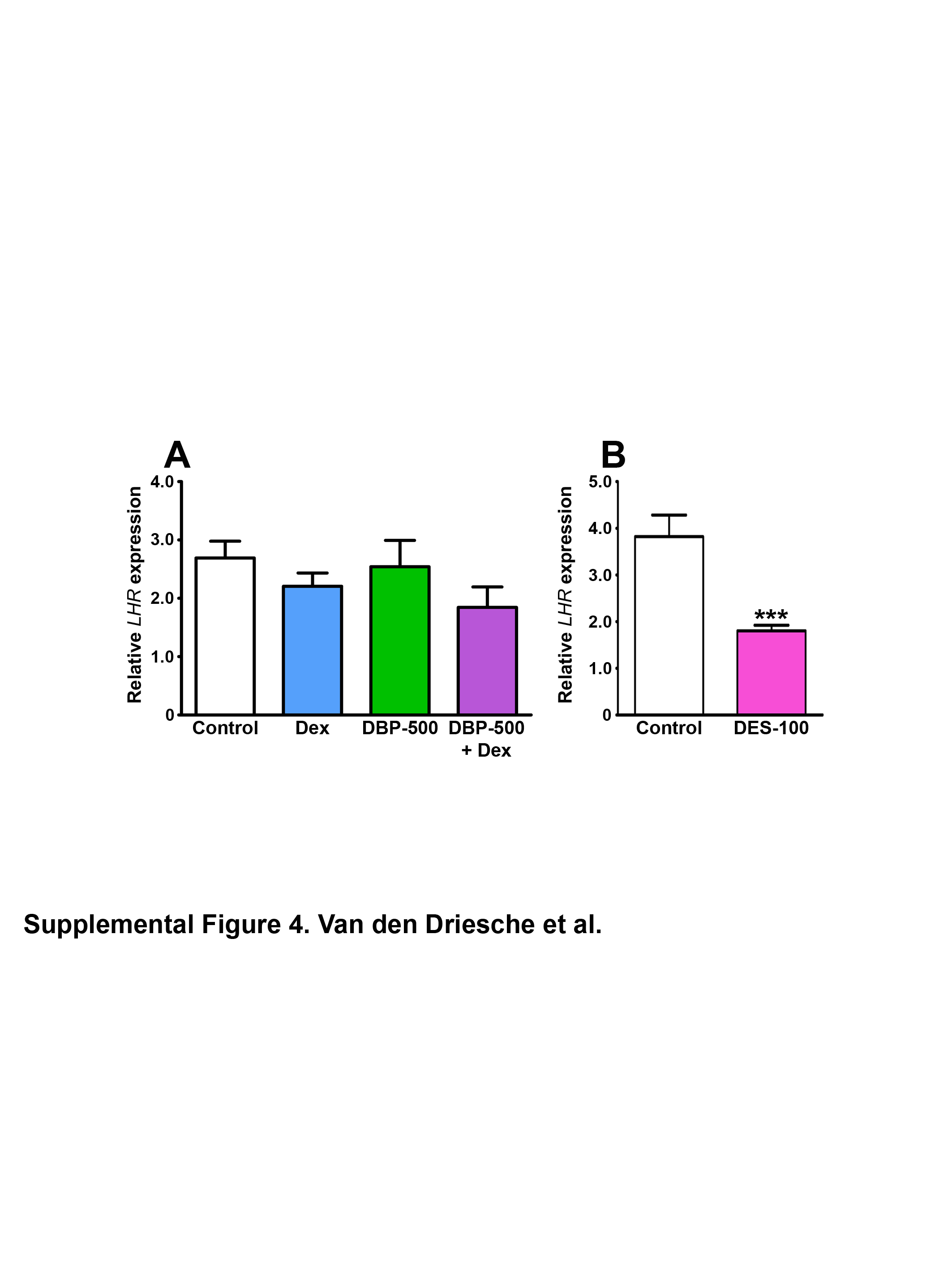

Supplement: Figure S4 — Effect of in utero exposure of rats to (A) vehicle (control), dexamethasone (Dex 100 µg/kg/day), dibutyl phthalate (DBP 500 mg/kg/day) or a combination of DBP-500 + Dex or (B) diethylstilbestrol (DES 100 µg/kg) on luteinizing hormone receptor ( LHR ) gene expression in testes at e21.5. Values are Means ± SEM for 11–24 animals per group (minimum of 3 litters per group). ***p<0.001, in comparison with respective control. (TIF) [file pone.0037064.s004.tif]

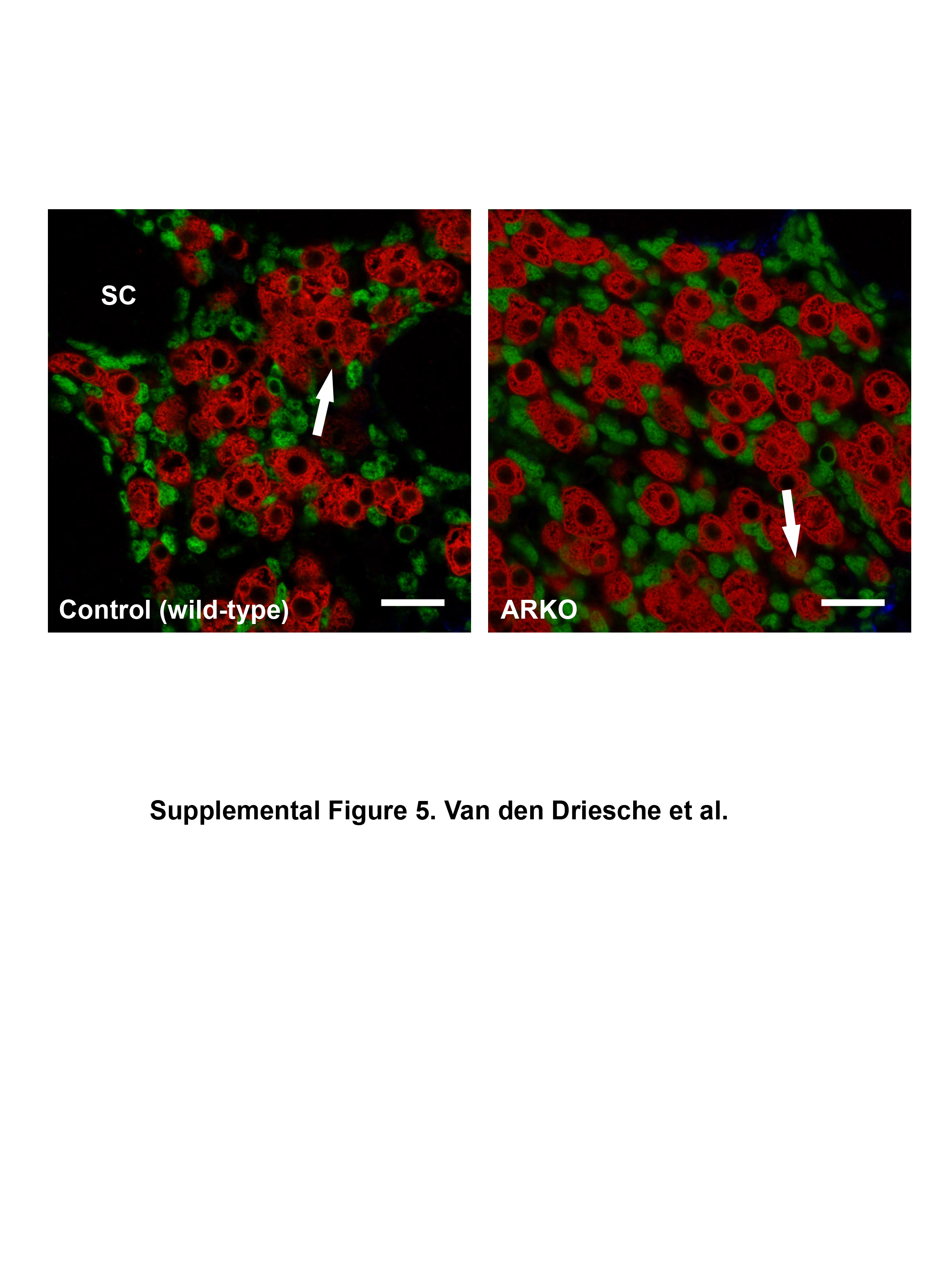

Supplement: Figure S5 — COUP-TFII immunoexpression in fetal LC in control and complete androgen receptor knockout (ARKO) mice at e18.5. Representative images of control and ARKO mice (n = 4) demonstrating that COUP-TFII is only rarely expressed (arrows) in ARKO fetal LC as in wild-type controls. Scale bar = 20 µm. (TIF) [file pone.0037064.s005.tif]

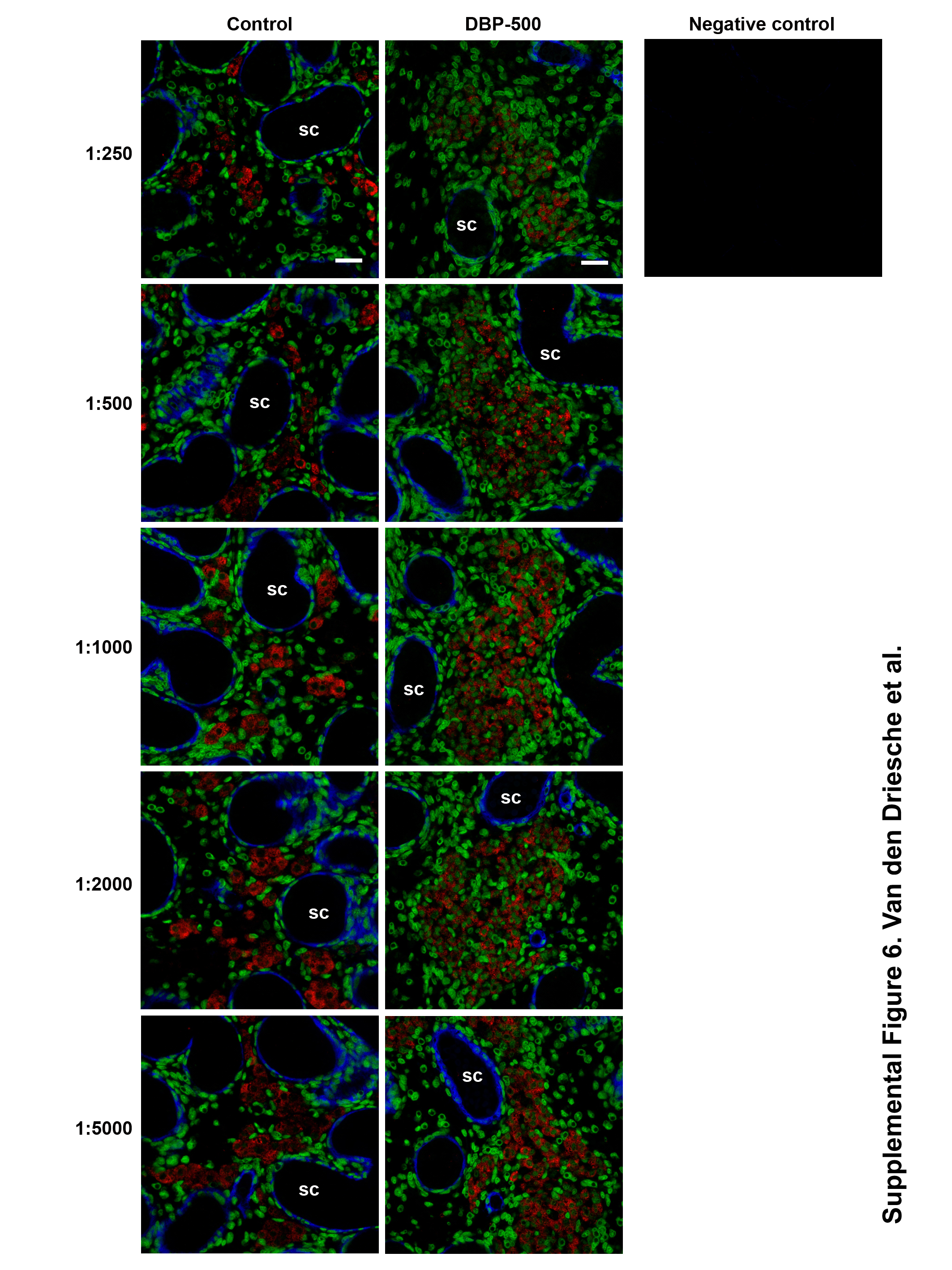

Supplement: Figure S6 — Serial dilution of COUP-TFII antibody. Triple immunofluorescence for SMA (blue), 3β-HSD (red) and COUP-TFII (green) on fetal testis sections from vehicle (control) and DBP-exposed (500 mg/kg/day) e21.5 animals. Note that in control sections most Leydig cells are COUP-TFII-immunopositive at low antibody dilutions (1∶250–1∶500) whereas only a minority is at lower antibody dilutions. In contrast, in sections from DBP-exposed animals, most Leydig cells are COUP-TFII-immunopositive at all antibody dilutions. SC = seminiferous cords. A wider range of antibody dilutions were run than is shown. Scale bar = 20 µm. (TIF) [file pone.0037064.s006.tif]
